# Supplementary material for: The Mia40 substrate Mix17 exposes its N-terminus to the cytosolic side of the mitochondrial outer membrane
Source: J Cell Sci. 2025 Apr 16;138(9):jcs263661. doi: 10.1242/jcs.263661 (PMC12045630; doi:10.1242/jcs.263661)
Supplement: Supplementary information [file joces-138-263661-s1.pdf]

```
H.sapiens        -----------------MPR--GSR-SAASRPASRPAAPSAHPPAHPPPSAAAPAPAPSG 40
R.norvegicus     -----------------MPR--GSR-SAAARPASRPA----HPPAHPPPSAPAPAPATSG 36
C.elegans        MVRRRTASPSPSAPVRSAPRPAAQSSFAAPPPRPAAAAPAYHPPAAPTP-MGAPMGAPSQ 59
N.crassa         MPRQSRGSARPSVPARK--------PVAPTNQQQQRPASTYAPPAAAPHAPPAAAAPVSQ 52
S.cerevisiae     -MARSRGSSRPISRSRPTQTRSASTMAAPVHPQQQQQPNAYS--------HPPAAGAQTR 51
                                            *                              :

H.sapiens        QPGLMAQMATTAAGVAVGSAVGHVMGSALTGAFSGGSSEPSQP-----AVQQAPTPAAPQ 95
R.norvegicus     QPGLMAQMASTAAGVAVGSAVGHVMGSALTSAFSGGSSEPAQP-----AVQQAPARPASH 91
C.elegans        GPGLMKQMAATAGGVAIGSAVGHAVGGMFTGGGSSH--AEQAP-----AAAAAPAGAPQA 112
N.crassa         GPGLFGQMASTAAGVAIGSSIGHAI----GGMFSGGGSSAAPEAAAAPVQ--AQAAAAQN 106
S.cerevisiae     QPGMFAQMASTAAGVAVGSTIGHTLGAGITGMFSGSGSDSAPVEQQQQNMANTSGQTQTD 111
                  **:: ***:**.***:**::**.:     .  *.                 :

H.sapiens        PLQMGPCAYEIRQFLDCST-TQSDLSLCEGFSEALKQCKYYHGLSSLP 142
R.norvegicus     PLQMGPCAYEIKQFLDCST-TQSDLTLCEGFSEALKQCKYNHGLSSLP 138
C.elegans        SGYSQPCEFEWRQFVDCAQ-NQSDVSLCNGFNDIFKQCKARYA----- 154
N.crassa         SSWGNNCSEATKSFTQCMDQHQGNMQICGWYLEQLKACQAAASQY--- 151
S.cerevisiae     QQLGRTCEIDARNFTRCLDENNGNFQICDYYLQQLKACQEAARQY*-- 156
                       *    :.*  *    :.:. :*  : : :* *:
```

Fig. S1. Mix17 is conserved throughout evolution

The amino acid sequence of *Saccharomyces cerevisiae* Mix17 and its orthologs in *Homo sapiens*, *Rattus norvegicus*, *Caenorhabditis elegans*, and *Neurospora crassa* were aligned using Clustal Omega (Madeira et al. 2022). Bolt, conserved hydrophobic segment; underlined, immunogenic peptide used for antibody generation; box, conserved CX_9C motif.

Journal of Cell Science • Supplementary information

Fig. 1A

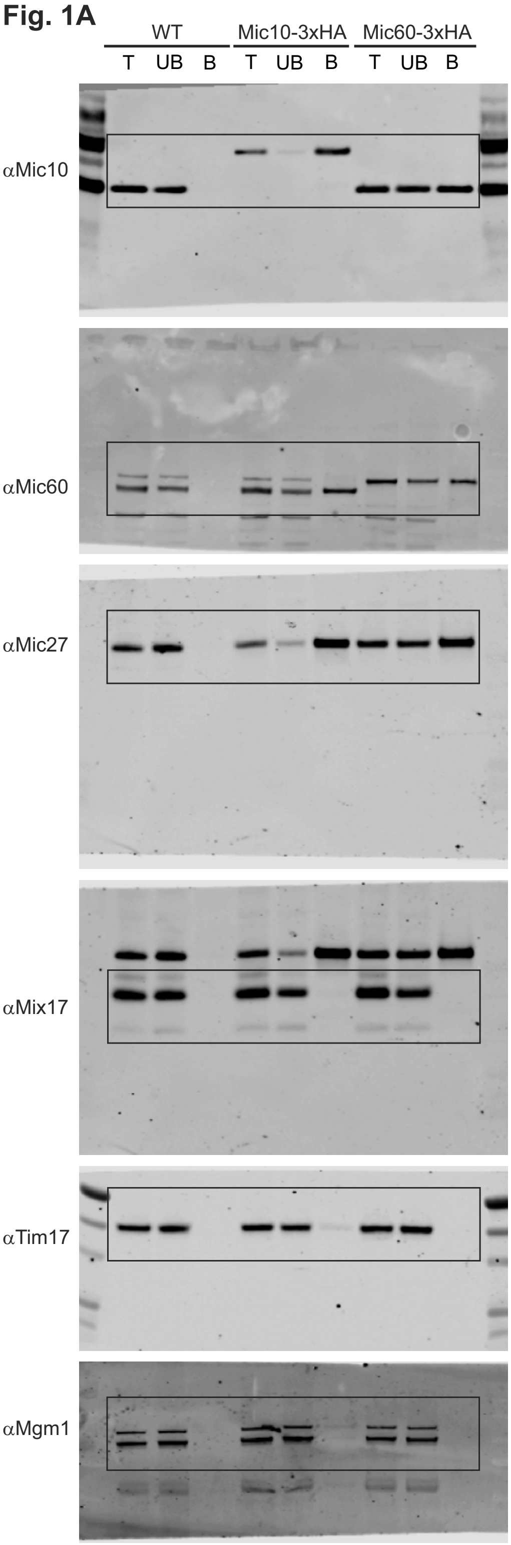

Fig. 1B

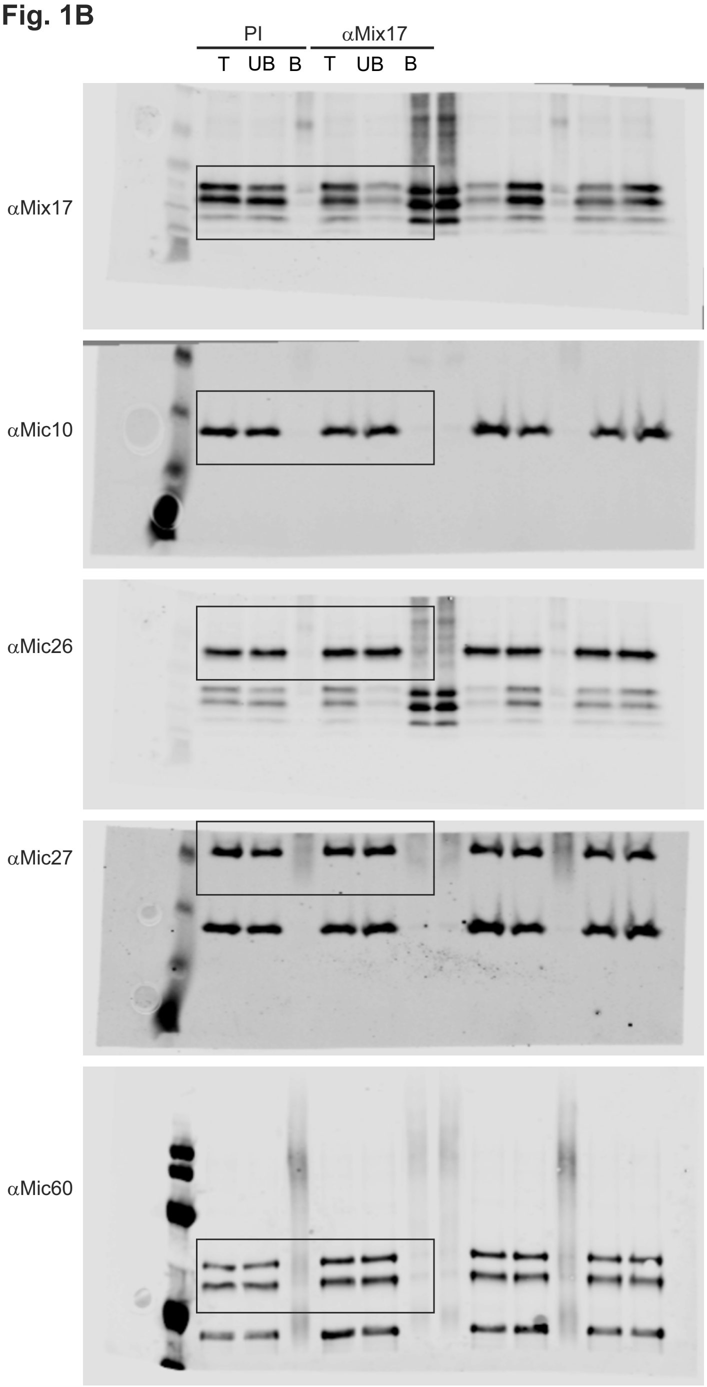

Journal of Cell Science • Supplementary information

Fig. 2A

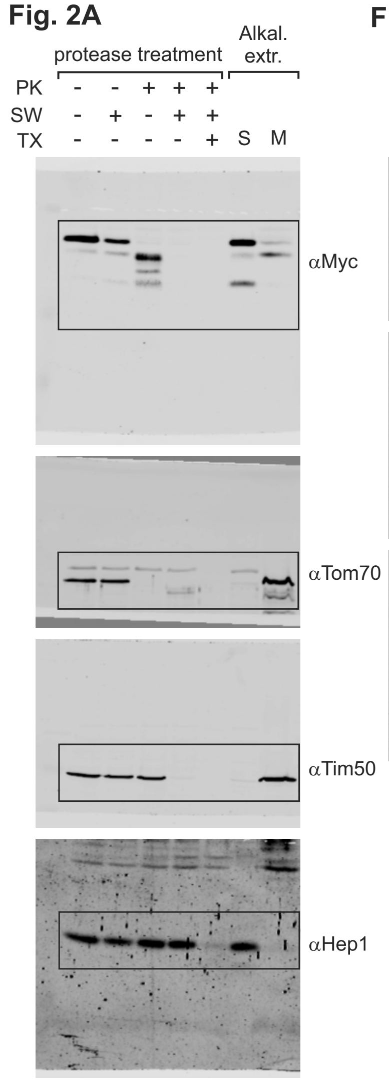

Fig. 2B

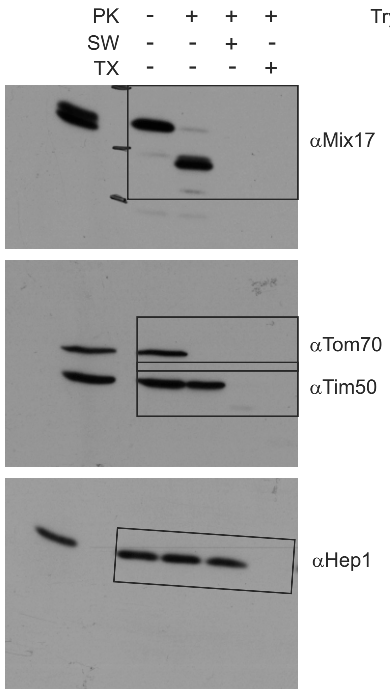

Fig. 2C

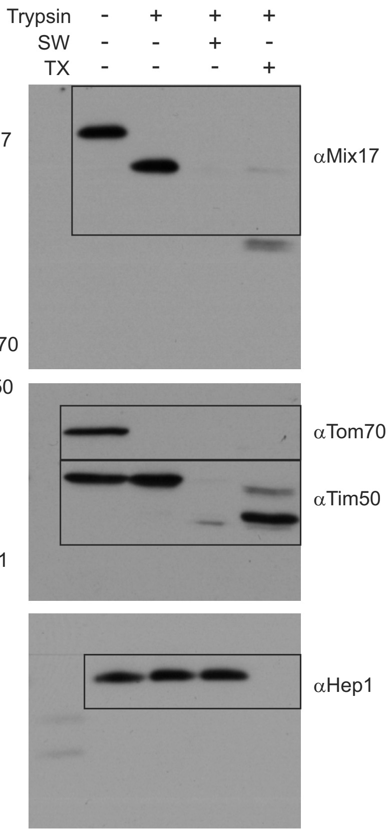

Fig. 2D

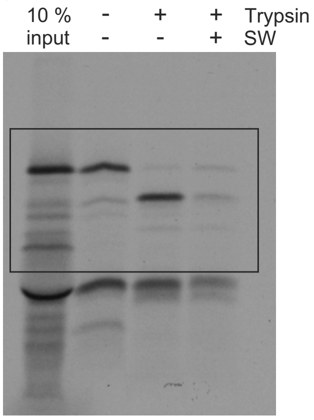

Journal of Cell Science • Supplementary information

Fig. 3A

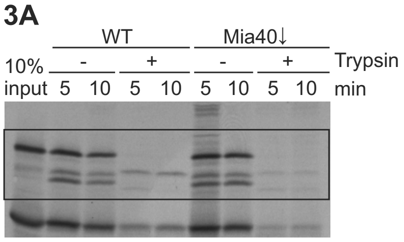

Fig. 3B

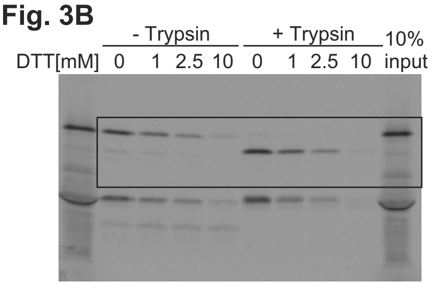

Fig. 3C

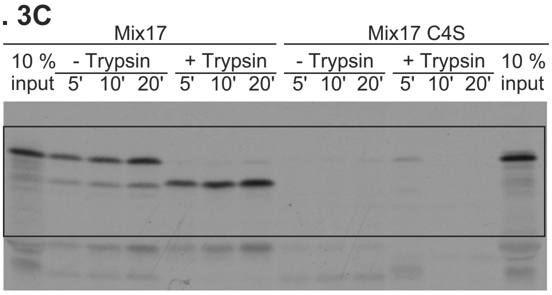

Fig. 3D

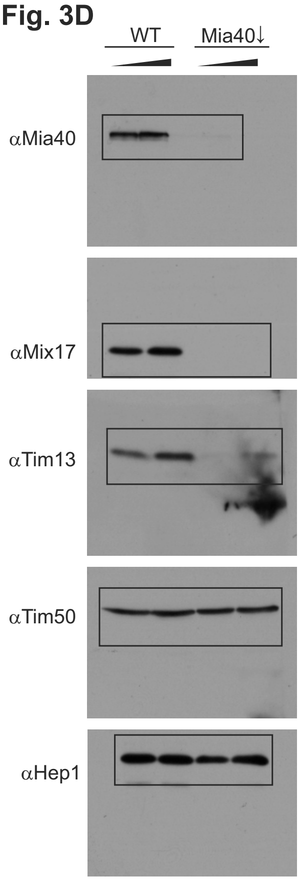

Fig. 4A

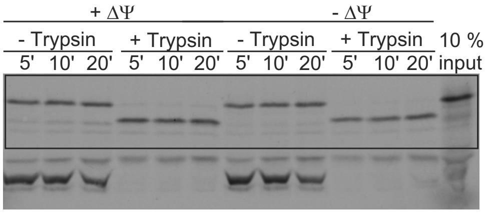

Fig. 4B

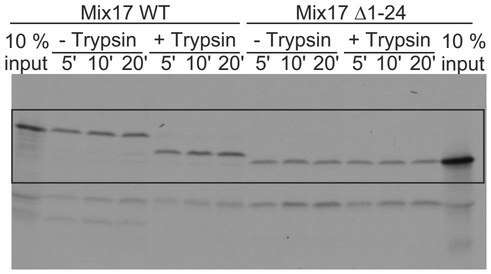

Fig. 4C

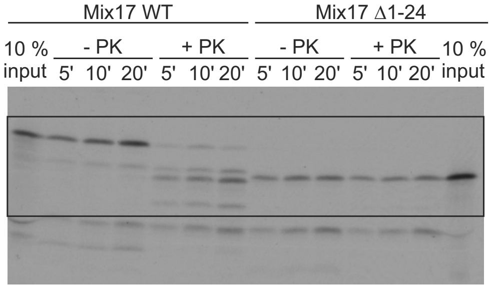

Fig. 4D

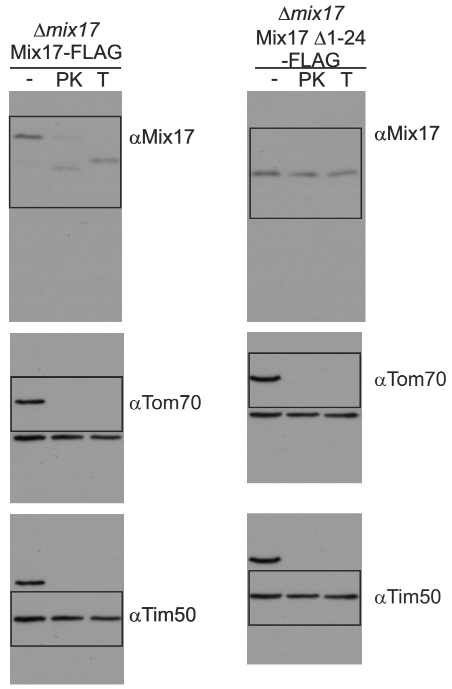

Journal of Cell Science • Supplementary information

Fig. 5

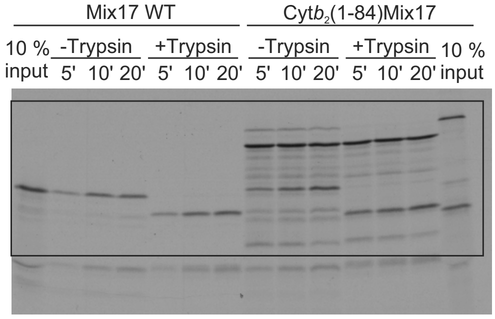

Journal of Cell Science • Supplementary information

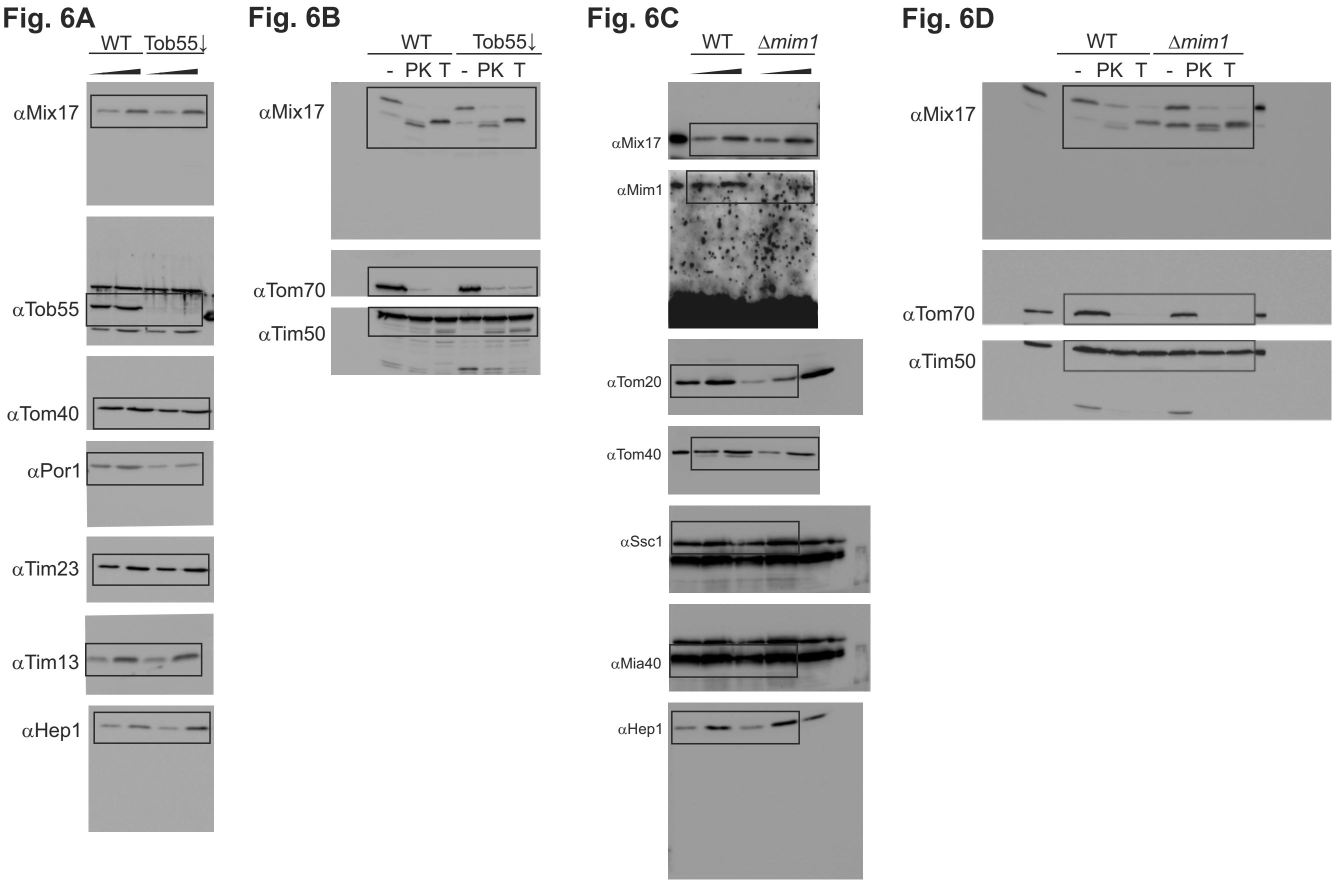
Fig. 6A
WT
Tob55↓
αMix17
αTob55
αTom40
αPor1
αTim23
αTim13
αHep1
Fig. 6B
WT
Tob55↓
- PK T - PK T
αMix17
αTom70
αTim50
Fig. 6C
WT
Δmim1
αMix17
αMim1
αTom20
αTom40
αSsc1
αMia40
αHep1
Fig. 6D
WT
Δmim1
- PK T - PK T
αMix17
αTom70
αTim50

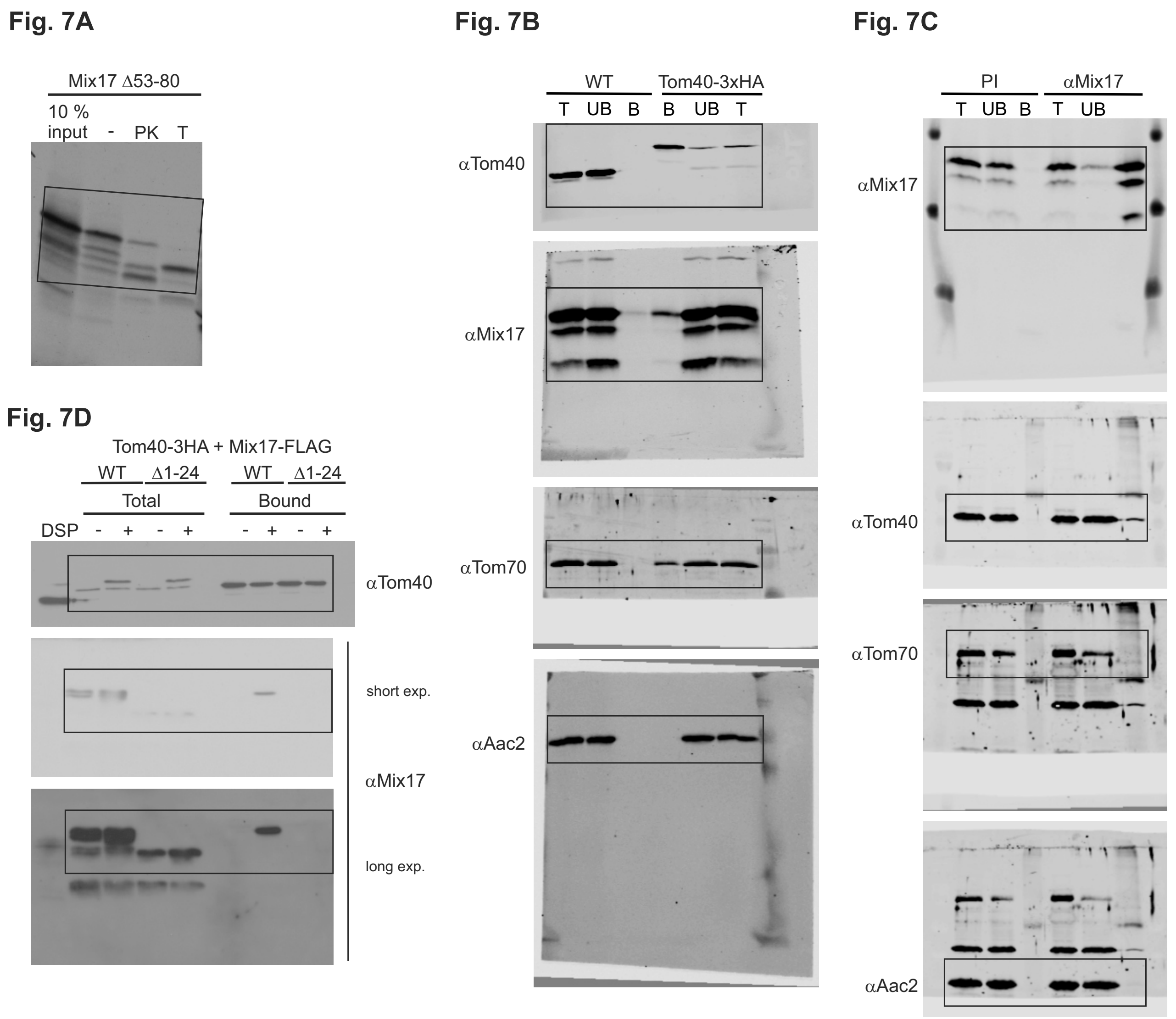

Fig. S2. Blot transparency

Journal of Cell Science • Supplementary information

Table S1. ***S. cerevisiae*** **strains used in this study.**

Strain name	Genotype	Reference
YPH499	*MAT*a *ade2-101 his3-Δ200 leu2- trp1-Δ63 ura3-52 lys2-801*	(Sikorski and Hieter 1989)
D273-10B	*MAT*α *mal GAL*	(Sherman 1963)
Mix17-3xMyc	YPH499 *MIX17::3xMyc::HIS3MX6*	This study
Tom40-3xHA	YPH499 *TOM40-3xHA::HIS3MX6*	This study
Mic10-3xHA	YPH499 *MIC10-3xHA::HIS3MX6*	(Harner et al. 2011)
Mic60-3xHA	YPH499 *MIC60-3xHA::HIS3MX6*	(Harner et al. 2011)
Δ*mix17*	YPH499 *mix17Δ::LEU2 (K. lactis)*	This study
Δ*mim1*	YPH499 *mim1Δ::LEU2 (K. lactis)*	This study
Mia40↓	YPH499 *kanMX4::P_{GAL10} MIA40*	(Terziyska et al. 2005)
Tob55↓	YPH499 *kanMX4::P_{GAL10} TOB55*	(Paschen et al. 2003)

Table S2. Vector constructs and primers used in this study.

Construct	Primer name	Restriction site	Sequence
pYX233 Mix17-FLAG	RI ACC Mix17	EcoRI	ctcgaattcaccatggcacgttcaagagg atcatc
	Mix17 XhoI nSt	XhoI	ctcctcgaggtattgacgtgcagcttcctgg caggc
pYX233 Mix17 Δ1-24-FLAG	RI ACC Mix17 Δ24	EcoRI	ctcgaattcaccatggcggctccagttca
	Mix17 XhoI nSt	XhoI	ctcctcgaggtattgacgtgcagcttcctgg caggc
pGEM4 Mix17C4S	Mix17 CS 1	-	aaactttacacgttctttggatgaaaacaac ggc
	Mix17 CS 2	-	ctcgcgtctatttcagaagttcttcccaactg ctg
	Mix17 CS 3	-	caactaaaagcctcccaggaagctgcac gtcaa
	Mix17 CS 4	-	tgcaagtaataatcagatatctggaagttg ccgttg
pGEM4 Mix17	pGEM4 Mix17_for	Xma1	gaatacacggaattcgagctcggtacccg ggatggcacgttcaagagg
	pGEM4 Mix17_rev	Pst1	tatagggagaccggaagcttgcatgcctg cagttagtattgacgtgcagc
	Mix17 M25A for	-	cgcttctaccgcggcggctccag
	Mix17 M25A rev	-	gaccttgtctgagtaggtc
pGEM4 Mix17 Δ1-24	pGEM4 Mix17Δ24_for	Xma1	gaatacacggaattcgagctcggtacccg ggtccgcttctaccatggc
	pGEM4 Mix17_rev	Pst1	tatagggagaccggaagcttgcatgcctg cagttagtattgacgtgcagc
pGEM4 Mix17 Δ53-80	Mix17 Δ53-80 f	-	accggtatgttttccggatc
	Mix17 Δ53-80 r	-	ctgtctcgtttgggcacc
pGEM4 Cyt*b*$_2$(1-84) Mix17	Mix17 XhoI	XhoI	ctcctcgagttagtattgacgtgcagcttcct g
	BglII Mix17	BglII	ctcagatctatggcacgttcaagaggatca tc
	KpnI Cyt*b*$_2$	KpnI	ctcggtacccgggatgctaaaatacaaac ctttactaaaaatc
	Mix17 HindIII	HindIII	gaccggaagcttgcatgcctgcagttagta ttgacgtgcagc

Journal of Cell Science • Supplementary information

Table S3. Antibodies used in this study.

Antibody	Source	Identifier/Reference	Dilution
IRDye 680RD Goat anti-Mouse IgG	LI-COR	Cat. #962-68070	1:10,000
IRDye 800CW Goat anti-Rabbit IgG	LI-COR	Cat. #926-32211	1:10,000
Goat Anti-Rabbit IgG-HRP Conjugate	BIO-RAD	Cat. #170-6515	1:10,000
Mouse monoclonal anti-Myc	Roche	Cat. #ROAMYC	1:250
Rabbit polyclonal anti-Mix17	LMU Munich	This study	1:250
Mouse monoclonal anti-HA	Santa Cruz	Cat. #sc-57592	1:250
Rabbit polyclonal anti-Tim13	LMU Munich	(Paschen et al. 2000)	1:200
Rabbit polyclonal anti-Tim17	LMU Munich	(Moro et al. 1999)	1:500
Rabbit polyclonal anti-Tim23	LMU Munich	(Mokranjac et al. 2003)	1:500
Rabbit polyclonal anti-Tim50	LMU Munich	(Mokranjac et al. 2003)	1:500
Rabbit polyclonal anti-Tom20	LMU Munich	(Krimmer et al. 2001)	1:500
Rabbit polyclonal anti-Tom40	LMU Munich	(Kiebler et al. 1990)	1:1,000
Rabbit polyclonal anti-Tom70	LMU Munich	(Schlossmann et al. 1996)	1:500
Rabbit polyclonal anti-Tob55	LMU Munich	(Paschen et al. 2003)	1:250
Rabbit polyclonal anti-Ssc1	LMU Munich	(Sichting et al. 2005)	1:1000
Rabbit polyclonal anti- Mia40	LMU Munich	(Terziyska et al. 2005)	1:500
Rabbit polyclonal anti- Mic10	LMU Munich	(Harner et al. 2011)	1:250
Rabbit polyclonal anti- Mic26	LMU Munich	(Harner et al. 2011)	1:250
Rabbit polyclonal anti- Mic27	LMU Munich	(Harner et al. 2011)	1:250
Rabbit polyclonal anti- Mic60	LMU Munich	(Rabl et al. 2009)	1:250
Rabbit polyclonal anti- Mgm1	LMU Munich	(Harner et al. 2016)	1:250
Rabbit polyclonal anti-Mim1	LMU Munich	(Waizenegger et al. 2005)	1:250
Rabbit polyclonal anti-Hep1	LMU Munich	(Sichting et al. 2005)	1:250
Rabbit polyclonal anti-Por1	LMU Munich	(Kleene et al. 1987)	1:1,000
Rabbit polyclonal anti-Aco1	LMU Munich	(Adam et al. 2006)	1:1,000
Rabbit polyclonal anti-Aac2	LMU Munich	(Sollner et al. 1990)	1:1,000

Supplementary References

Adam, A. C., C. Bornhovd, H. Prokisch, W. Neupert and K. Hell (2006). The Nfs1 interacting protein Isd11 has an essential role in Fe/S cluster biogenesis in mitochondria. EMBO J *25*, 174-183. 10.1038/sj.emboj.7600905.

Harner, M., C. Korner, D. Walther, D. Mokranjac, J. Kaesmacher, U. Welsch, J. Griffith, M. Mann, F. Reggiori and W. Neupert (2011). The mitochondrial contact site complex, a determinant of mitochondrial architecture. EMBO J *30*, 4356-4370. 10.1038/emboj.2011.379.

Harner, M. E., A. K. Unger, W. J. Geerts, M. Mari, T. Izawa, M. Stenger, S. Geimer, F. Reggiori, B. Westermann and W. Neupert (2016). An evidence based hypothesis on the existence of two pathways of mitochondrial crista formation. Elife *5*. 10.7554/eLife.18853.

Kiebler, M., R. Pfaller, T. Sollner, G. Griffiths, H. Horstmann, N. Pfanner and W. Neupert (1990). Identification of a mitochondrial receptor complex required for recognition and membrane insertion of precursor proteins. Nature *348*, 610-616.

Kleene, R., N. Pfanner, R. Pfaller, T. A. Link, W. Sebald, W. Neupert and M. Tropschug (1987). Mitochondrial porin of Neurospora crassa: cDNA cloning, in vitro expression and import into mitochondria. EMBO J *6*, 2627-2633. 10.1002/j.1460-2075.1987.tb02553.x.

Krimmer, T., D. Rapaport, M. T. Ryan, C. Meisinger, C. K. Kassenbrock, E. Blachly-Dyson, M. Forte, M. G. Douglas, W. Neupert, F. E. Nargang and N. Pfanner (2001). Biogenesis of porin of the outer mitochondrial membrane involves an import pathway via receptors and the general import pore of the TOM complex. J Cell Biol *152*, 289-300. 10.1083/jcb.152.2.289.

Madeira, F., M. Pearce, A. R. N. Tivey, P. Basutkar, J. Lee, O. Edbali, N. Madhusoodanan, A. Kolesnikov and R. Lopez (2022). Search and sequence analysis tools services from EMBL-EBI in 2022. Nucleic Acids Res *50*, W276-W279. 10.1093/nar/gkac240.

Journal of Cell Science • Supplementary information

Mokranjac, D., S. A. Paschen, C. Kozany, H. Prokisch, S. C. Hoppins, F. E. Nargang, W. Neupert and K. Hell (2003). Tim50, a novel component of the TIM23 preprotein translocase of mitochondria. EMBO J. *22*, 816-825. 10.1093/emboj/cdg090.
Moro, F., C. Sirrenberg, H. C. Schneider, W. Neupert and M. Brunner (1999). The TIM17.23 preprotein translocase of mitochondria: composition and function in protein transport into the matrix. EMBO J. *18*, 3667-3675. 10.1093/emboj/18.13.3667.
Paschen, S. A., U. Rothbauer, K. Kaldi, M. F. Bauer, W. Neupert and M. Brunner (2000). The role of the TIM8-13 complex in the import of Tim23 into mitochondria. EMBO J *19*, 6392-6400. 10.1093/emboj/19.23.6392.
Paschen, S. A., T. Waizenegger, T. Stan, M. Preuss, M. Cyrklaff, K. Hell, D. Rapaport and W. Neupert (2003). Evolutionary conservation of biogenesis of beta-barrel membrane proteins. Nature *426*, 862-866. 10.1038/nature02208.
Rabl, R., V. Soubannier, R. Scholz, F. Vogel, N. Mendl, A. Vasiljev-Neumeyer, C. Korner, R. Jagasia, T. Keil, W. Baumeister, M. Cyrklaff, W. Neupert and A. S. Reichert (2009). Formation of cristae and crista junctions in mitochondria depends on antagonism between Fcj1 and Su e/g. J Cell Biol *185*, 1047-1063. 10.1083/jcb.200811099.
Schlossmann, J., R. Lill, W. Neupert and D. A. Court (1996). Tom71, a novel homologue of the mitochondrial preprotein receptor Tom70. J. Biol. Chem. *271*, 17890-17895. 10.1074/jbc.271.30.17890.
Sherman, F. (1963). Respiration-deficient mutants of yeast. I. Genetics. Genetics *48*, 375-385. 10.1093/genetics/48.3.375.
Sichting, M., D. Mokranjac, A. Azem, W. Neupert and K. Hell (2005). Maintenance of structure and function of mitochondrial Hsp70 chaperones requires the chaperone Hep1. EMBO J *24*, 1046-1056. 10.1038/sj.emboj.7600580.
Sikorski, R. S. and P. Hieter (1989). A system of shuttle vectors and yeast host strains designed for efficient manipulation of DNA in Saccharomyces cerevisiae. Genetics *122*, 19-27. 10.1093/genetics/122.1.19.
Sollner, T., R. Pfaller, G. Griffiths, N. Pfanner and W. Neupert (1990). A mitochondrial import receptor for the ADP/ATP carrier. Cell *62*, 107-115. 0092-8674(90)90244-9 [pii].
Terziyska, N., T. Lutz, C. Kozany, D. Mokranjac, N. Mesecke, W. Neupert, J. M. Herrmann and K. Hell (2005). Mia40, a novel factor for protein import into the intermembrane space of mitochondria is able to bind metal ions. FEBS Lett *579*, 179-184. 10.1016/j.febslet.2004.11.072.
Waizenegger, T., S. Schmitt, J. Zivkovic, W. Neupert and D. Rapaport (2005). Mim1, a protein required for the assembly of the TOM complex of mitochondria. EMBO Rep *6*, 57-62. 10.1038/sj.embor.7400318.

Journal of Cell Science • Supplementary information
