# Supplementary material for: The Mia40 substrate Mix17 exposes its N-terminus to the cytosolic side of the mitochondrial outer membrane
Source: J Cell Sci. 2025 Apr 16;138(9):jcs263661. doi: 10.1242/jcs.263661 (PMC12045630; doi:10.1242/jcs.263661)
Supplement: Supplementary file 1 [file joces-138-263661_review_history.pdf]

Journal of Cell Science

The Mia40 substrate Mix17 exposes its N-terminus to the cytosolic side of the mitochondrial outer membrane

Moritz Resch, Johanna S. Frickel, Korbinian Dischinger, Rachel Shen Wen Choo, Kai Hell and Max E. Harner
DOI: 10.1242/jcs.263661

Editor: Jennifer Lippincott-Schwartz

Review timeline
Original submission: 3 June 2024
Editorial decision: 13 August 2024
First revision received: 29 January 2025
Accepted: 3 March 2025

Original submission

First decision letter

MS ID#: JOCES/2024/262318

MS TITLE: The N-terminus of mitochondrial Mix17 is exposed to the cytosol in an energy dependent manner

AUTHORS: Moritz Resch, Johanna S. Frickel, Rachel Choo Shen Wen, Kai G Hell, and Max E. Harner
ARTICLE TYPE: Research Article

Dear Dr. Harner,

We have now reached a decision on the above manuscript.

To see the reviewers' reports and a copy of this decision letter, please go to: https://submit-jcs.biologists.org and click on the 'Manuscripts with Decisions' queue in the Author Area. (Corresponding author only has access to reviews.)

Reviewer 1

Advance summary and potential significance to field

In this manuscript, Resch et al conducted an excellent analysis of the subcellular localization and biogenesis of a previously identified protein Mix17. While initially it was suggested that Mix17 is a typical Mia40 substrate, this study shows that the protein partially inserts into the outer membrane, without involvement of the TOB/SAM complex. Instead it reaches the outer membrane/IMS orientation taking a “conservative” import rout by translocation across the inner membrane followed by insertion into the outer membrane. Thus the biogenesis of Mix17 involves interaction with TOM40, TIM23 and finally Mia40. This a unique import process with a unique importance for the researchers in the field and the general reader. Over all the work is carefully conducted. I have two minor notes.

© 2025. Published by The Company of Biologists under the terms of the Creative Commons Attribution License (https://creativecommons.org/licenses/by/4.0/).

1) in Figure 2A the description of changes as drastic change. To make it obvious quantitation should be conducted.
2) A scheme or diagram depicting the import rout of Mix17 and its final interactions should probably be included.

Comments for the author

1) in Figure 2A the description of changes as drastic change. To make it obvious quantitation should be conducted.
2) A scheme or diagram depicting the import rout of Mix17 and its final interactions should probably be included.

Reviewer 2

Advance summary and potential significance to field

Mitochondrial biogenesis depends on the import of most mitochondrial proteins from the cytosol. Pathways and components for this mitochondrial protein import have been extensively studies, but we still do not have a complete list of which mitochondrial proteins use which pathways, relying on which components, for their import. Now in this manuscript, the authors report that yeast Mix17 is a novel substrate for the MIA pathway, which is typically used for small Cys-rich proteins in the intermembrane space (IMS), as Mix17 is partially inserted into the outer membrane (OM) from the IMS. This is interesting, but many key questions remain unanswered.
(1) What is the role of ATP? Is ATP used outside the mitochondria or inside? Is Mitochondrial Hsp70 (mtHsp70 or Ssc1 in yeast) involved in this ATP requirement, even though it is difficult to imagine that mtHsp70 in the matrix facilitates insertion of Mix17 into the OM from the IMS? These questions can be experimentally tested.
(2) What is the role of the interaction between Mix17 and Tom40? Although the authors showed that Tom40 IP could pull down Mix17, but what about the Mix17 pull-down? Perhaps, Mix17 is sticky to many proteins? If Mix17 interacts with Tom40 to penetrate to the other side of the OM, is Mix17 inserted into the b-barrel pore of Tom40? Since Mix17 apparently stays bound to Tom40 and is not integrated into the membrane environment, is Mix17 permanently inserted into the Tom40 pore? If so, does this interfere with the import of mitochondrial proteins through the Tom40 import channel? Perhaps, a more thorough crosslinking approach is required to determine whether Mix17 is inside the Tom40 pore or inserted into the lipid-protein interface of Tom40.
Although it will take more time to address all the points mentioned above, conducting some of these experiments could strengthen the manuscript. In addition, the authors can also discuss these points in the Discussion section.

Comments for the author

As I wrote above, the authors do not have to perform experiments to meet all the above points.

Reviewer 3

Advance summary and potential significance to field

Harner and co-workers present a very interesting study on the biogenesis of an unusually behaving mitochondrial intermembran

Comments for the author

© 2025. Published by The Company of Biologists under the terms of the Creative Commons Attribution License (https://creativecommons.org/licenses/by/4.0/).

Major points:
- It remains unclear in yeast Mix17 also interacts with MICOS, like its mammalian ortholog obviously does. The authors shoul
- The mode of interaction with the TOM complex needs further clarification. For example, does Mix17 occupy part of the prote
- The authors state that the outer surface exposure of the Mix17 N-terminus is energy-dependent, however the experiment in F Minor point:
Figure 1C: Why is Tim50 fully degraded upon osmotic swelling, but only partially cleaved by trypsin in TX-100? Isn't this su

---

First revision

Author response to reviewers' comments

We want to thank you and the three reviewers for the careful evaluation and the thoughtful suggestions and comments. In the following we address point-by-point the comments and suggestions raised by the reviewers.

Reviewer 1 Advance Summary and Potential Significance to Field:
In this manuscript, Resch et al conducted an excellent analysis of the subcellular localization and biogenesis of a previously identified protein Mix17. While initially it was suggested that Mix17 is a typical Mia40 substrate, this study shows that the protein partially inserts into the outer membrane, without involvement of the TOB/SAM complex. Instead it reaches the outer membrane/IMS orientation taking a “conservative” import rout by translocation across the inner membrane followed by insertion into the outer membrane. Thus the biogenesis of Mix17 involves interaction with TOM40, TIM23 and finally Mia40. This a unique import process with a unique importance for the researchers in the field and the general reader. Over all the work is carefully conducted. I have two minor notes.
We thank the reviewer 1 for his/her thoughtful comments.

1) in Figure 2A the description of changes as drastic change. To make it obvious quantitation should be conducted.
We agree with the reviewer. The requested quantification has been added in the new **Fig. 3**.

2) A scheme or diagram depicting the import rout of Mix17 and its final interactions should probably be included.
We now added a model in the new **Fig. 8** indicating the relevance of the Mix17 N-terminus for its import and exposure to the cytosolic side of the outer membrane. The model further suggests two conceivable locations of Mix17 in close proximity to Tom40, within the protein-conducting channel or at the lipid interface of Tom40.

Reviewer 2 Advance Summary and Potential Significance to Field:
Mitochondrial biogenesis depends on the import of most mitochondrial proteins from the cytosol. Pathways and components for this mitochondrial protein import have been extensively studies, but we still do not have a complete list of which mitochondrial proteins use which pathways, relying on which components, for their import. Now in this manuscript, the authors report that yeast Mix17 is a novel substrate for the MIA pathway, which is typically used for small Cys-rich proteins in the intermembrane space (IMS), as Mix17 is partially inserted into the outer membrane (OM) from the IMS. This is interesting, but many key questions remain unanswered.
(1) What is the role of ATP? Is ATP used outside the mitochondria or inside? Is Mitochondrial Hsp70 (mtHsp70 or Ssc1 in yeast) involved in this ATP requirement, even though it is difficult to imagine that mtHsp70 in the matrix facilitates insertion of Mix17 into the OM from the IMS? These questions can be experimentally tested.

© 2025. Published by The Company of Biologists under the terms of the Creative Commons Attribution License (https://creativecommons.org/licenses/by/4.0/).

We thank the reviewer 2 for his/her thoughtful comments.

Our data clearly show that the N-terminus of Mix17 is exposed to the cytosolic side of the mitochondrial outer membrane. However, during the course of the revision we realized that the conditions determining the topology of Mix17 are much more complex than initially manifested. Thus, we feel that it is too early to attribute topology changes to ATP present in mitochondria and need more time to address this question. Consequently, we prefer to invest this interesting phenomenon in more detail before we draw final conclusions. We actually plan to do this in a future study. Therefore, we removed panel E from Fig. 1 (now Fig.2) in the revised manuscript and ask this reviewer for his/her understanding. Of course, we made the respective changes in title, abstract and main text sections.

(2) What is the role of the interaction between Mix17 and Tom40? Although the authors showed that Tom40 IP could pull down Mix17, but what about the Mix17 pull-down? Perhaps, Mix17 is sticky to many proteins? If Mix17 interacts with Tom40 to penetrate to the other side of the OM, is Mix17 inserted into the b-barrel pore of Tom40? Since Mix17 apparently stays bound to Tom40 and is not integrated into the membrane environment, is Mix17 permanently inserted into the Tom40 pore? If so, does this interfere with the import of mitochondrial proteins through the Tom40 import channel? Perhaps, a more thorough crosslinking approach is required to determine whether Mix17 is inside the Tom40 pore or inserted into the lipid-protein interface of Tom40.

The reviewer is absolutely right that the interaction between Mix17 and Tom40 requires a more detailed analysis. We showed that immunoprecipitated Tom40 co-isolated Mix17 after treatment of mitochondria with crosslinking reagent. As suggested by the reviewer, we now also immunoprecipitated Mix17 with a Mix17-specific antibody and were able to co-isolate Tom40 from crosslinked mitochondria. In contrast, the ADP/ATP carrier Aac2 or Tom70 could not be co-isolated indicating the specificity of this interaction. These results show that the co-isolation of Mix17 and Tom40 can be obtained from both sides, further supporting the specificity of this interaction. Since Mix17 crosslinked to Tom40, but not to Tom70, the data may suggest that Mix17 is in close proximity to the Tom40 pore of the TOM complex rather than to the Tom70 receptor. We included these additional data in the new **Fig. 7C**.
We agree with the reviewer that a sophisticated crosslinking approach, such as the one employed by Shiota et al. in 2011 and 2015, is required to analyze whether Mix17 inserts in the β-barrel pore of Tom40 or whether it is present at the lipid-protein interface of Tom40. We believe that such a complex crosslinking approach is beyond the scope of this study, since it requires modified protein variants and well-established experience with the method.
However, we addressed the important point of the reviewer whether Mix17 would affect the import of proteins into mitochondria, if inserted in the Tom40 pore. In case of interference of Mix17 insertion in the pore with import into mitochondria, we hypothesized that overexpression of Mix17 would increase such an effect (see also reviewer 3). To test it, we made use of our strain encoding *MIX17-FLAG* under the control of the strong GAL promoter. Induction of gene expression for 1.5 h prior to mitochondrial preparation resulted in a ca. 8x overexpression of Mix17-FLAG compared to wild type Mix17 (**Fig. 1A for the reviewers**). According to available mass spectrometry data, this should result in comparable protein levels of Mix17 and Tom40 (Nagaraj et al. 2012, Kulak et al. 2014). Next, we tested the topology of overexpressed Mix17-FLAG. Virtually all Mix17-FLAG was accessible to PK and trypsin, indicating that the N-terminus is still exposed to the cytosolic side of the outer membrane (**Fig. 1B for thereviewers**). Finally, we analyzed the import efficiency of these mitochondria compared to a wild type control. Overexpression of *MIX17* did not impair the import of the Mia40 substrate Tim10 and the TIM23 substrate Su9-DHFR (**Fig. 1C,D for the reviewers**). Together, this indicates that overexpression of *MIX17* does not disturb the protein import into mitochondria.
However, before one can draw a conclusion about the interference between pore-inserted Mix17 and mitochondrial import, it requires further experimentation, such as detailed cross-linking, to discriminate whether (i) Mix17 is present in the pore, (ii) whether it inserts in the pore in a dynamic manner or (iii) whether it is not present in the pore at all. We would need to be sure that

© 2025. Published by The Company of Biologists under the terms of the Creative Commons Attribution License (https://creativecommons.org/licenses/by/4.0/).

all TOM complexes could be blocked. Therefore, we prefer not to add the experiments shown in **Fig. 1 for the reviewers** to the manuscript at this stage.

We have removed a figure that was provided for the referees in confidence.

Fig. 1 for the reviewers. Overexpression of MIX17 does not disturb the mitochondrial protein import.
(A) Galactose induced gene expression results in significant overexpression of *MIX17-FLAG*. A yeast strain harboring a plasmid to overexpress MIX17-FLAG or a wild type control were both cultured on synthetic medium containing glucose as carbon source. Gene expression was induced by the addition of galactose for 1.5 h prior to mitochondrial preparation. The indicated amounts of mitochondrial proteins were loaded on a SDS-gel and proteins levels were analyzed by SDS-PAGE and immunoblotting. **(B)** Overexpressed Mix17-FLAG is still accessible to proteases in intact mitochondria. Mitochondria from the Mix17-FLAG overexpression strain and a wild type control were subjected to the proteases trypsin and proteinase K (PK). Samples were analyzed by SDS-PAGE and immunoblotting. f (T), f (PK), fragments generated by trypsin and PK, respectively. **(C,D)** The imports of Tim10 and Su9-DHFR are not disturbed upon overexpression of Mix17. ^{35}S-labeled Tim10 (C) or pSu9-DHFR (D) were incubated with mitochondria from the *MIX17-FLAG* overexpression strain or a wild type control. Samples were taken at the indicated time points. Subsequently, the samples with Tim10 were incubated without PK (-PK) or with PK (50µg/ml) to remove non-imported Tim10 precursor. Samples with pSu9-DHFR were treated with PK. Samples were analyzed by SDS-PAGE and autoradiography. (Upper panels) Representative autoradiographs are shown. pSu9-DHFR, precursor of a fusion protein consisting of the N-terminal 69 residues of the presubunit 9 of ATP synthase and dihydrofolate reductase; m, mature form of pSu9-DHFR after import and cutting off the presequence (Lower panels) Quantifications of the respective experiments are shown. PK-protected material of Tim10 and the mature form of pSu9-DHFR were quantified. Results are presented as means of the imported protein at the indicated time points normalized to the maximal imported protein (100%). Error bars, standard deviation.

Reviewer 3 Advance Summary and Potential Significance to Field:
Harner and co-workers present a very interesting study on the biogenesis of an unusually behaving mitochondrial intermembrane space protein in yeast, termed Mix17. In contrast to earlier reports, the authors clearly show that Mix17 is partially exposed to the outer mitochondrial surface, likely via the TOM complex and in an energy-dependent manner. I am impressed by the outstanding quality of the experimental data and by the stringent chain of reasoning in this manuscript. Although the study may be primarily stimulating for experts in the field of mitochondrial biogenesis, it paves the ground for further in-depth functional analysis of an exciting two-membrane-spanning, maybe energy-state-sensing mechanism connected to mitochondrial membrane contact sites and cristae. I strongly recommend the manuscript for publication in the Journal of Cell Science in principle, after the authors have addressed the following questions.

Major points:

- It remains unclear in yeast whether Mix17 also interacts with MICOS, like its mammalian ortholog obviously does. The authors should clarify this point! (Although it would also be acceptable for me, if they wish to keep it for a separate study on the novel subunit of yeast MICOS. The present work is strong and substantial enough as it stands.)
We thank the reviewer for his/her thoughtful comments. We agree that it needs to be clarified whether Mix17 interacts with MICOS in yeast. To this end, we performed co-immunoprecipitation assays. We did not detect an interaction of Mix17 and MICOS independently whether we pulled on Mix17 or the MICOS subunits Mic10 and Mic60. As positive control, other MICOS subunits were co-precipitated with Mic10 and Mic60. Based on these results, we conclude, that Mix17 appears not to be associated with MICOS in yeast. Of note, the interaction of Mix17 and MICOS in the human system has been proposed to be cell type specific (Burstein et al. 2018). We cannot exclude that yeast Mix17 and MICOS might also only interact under certain conditions. Thus, we added our data in a new **Fig. 1**, and discussed this finding rather carefully in the manuscript.

© 2025. Published by The Company of Biologists under the terms of the Creative Commons Attribution License (https://creativecommons.org/licenses/by/4.0/).

- The mode of interaction with the TOM complex needs further clarification. For example, does Mix17 occupy part of the protein-conducting channel(s) of TOM? It is possible to estimate, which percentage of TOM complexes contain Mix17 (under fully energized conditions)? Does any of the recently established TOM clogging constructs interfere with Mix17-TOM association? Or does overexpression of Mix17 interfere with TOM-dependent protein import?

Reviewer 3 is addressing an important point here. We highly appreciate the valuable questions of reviewer 3. Regarding the question whether Mix17 sits in the Tom40 pore, we presented first data, which are consistent with this notion: 1. Endogenous Mix17 can be crosslinked and then co-isolated with Tom40. 2. Mix17 spans the mitochondrial outer membrane most likely in a proteinaceous environment. s pointed out in the response to **Reviewer 2** point 2, sophisticated and lengthy crosslinking approaches, however, would be required to analyze this interaction in detail. These approaches are, at present, not possible for us in the timeline of this study.

In our opinion, it is not possible to estimate the amount of TOM complexes interacting with Mix17. We show in **Fig. 6D (new Fig. 7D)** that the association of Mix17 and Tom40 requires DSP crosslinking. The cross-linking approach is rather a qualitative, but not a quantitative technique to analyze interactions and, thus, not suited for a quantitative calculation of an interaction. In addition, we would need solid data on the amount of Mix17 and Tom40 molecules per cell for an estimation. Indeed, these amounts have been analyzed in various studies by quantitative mass spectrometry (Picotti et al. 2009, Nagaraj et al. 2012, Kulak et al. 2014). However, the reported data vary quite strongly, ranging from rather equimolar ratio (Picotti et al. 2009) to an almost 10x excess of Tom40 relative to Mix17 (Nagaraj et al. 2012, Kulak et al. 2014). Moreover, it is also unclear, how many Mix17 molecules would be able to insert in one pore of the TOM complex, if Mix17 stays permanently in the pore.

As suggested by the reviewer, we analyzed the effect of overexpression of *MIX17* on the mitochondrial protein import. To this end, we decided to use a strain expressing *MIX17* under the control of the strong GAL promotor, resulting in a roughly 8-fold increase in Mix17 (**Fig. 1A for the reviewers**). Thus, assuming an 10-fold excess of Tom40 relative to Mix17, we would expect a significant effect of 8-fold overexpression of Mix17 on protein import, if Mix17 were to interfere with TOM-dependent protein import. Interestingly, although Mix17 is still exposed to the cytosol under these conditions (**Fig. 1B for the reviewers**), the import of the precursor proteins, Su9-DHFR and Tim10, was not inhibited (**Fig. 1C,D for the reviewers**). Please see also our response to reviewer 2 point 2.

Finally, we followed this reviewer's suggestion and analyzed the interaction between Mix17 and Tom40 in the presence of the established clogging construct cytochrome b_2(167)ΔDHFR (generous gift from Dr. Dejana Mokranjac). Methotrexate treatment of recombinantly expressed cytochrome b_2(167)ΔDHFR prior to the import reaction results in a stable folding of the DHFR moiety of the fusion construct and, thus, in the inhibition of complete import of the fusion protein and in the formation of TIM23-TOM supercomplexes (Dekker et al. 1997, Chacinska et al. 2003, Mokranjac et al. 2005, Waegemann et al. 2015). Consequently, the TOM complexes are clogged (Dekker et al. 1997). When we isolated Tom40 in presence and absence of cytochrome b_2(167)ΔDHFR, we first saw a strongly increased amount of Tim23 co-purifying with Tom40 indicating the successful formation of TIM23-TOM supercomplexes (**Fig. 2 for the reviewers**). In addition, we detected a reduced co-isolation of Mix17 after import of chemical amounts of cytochrome b_2(167)ΔDHFR compared to control conditions. However, we realized that the efficiency of Tom40-3xHA immunoprecipitation was reproducibly lower upon cytochrome b_2(167)ΔDHFR import (**Fig. 2 for the reviewers**), likely due to sterical hindrance. When we quantified the co-isolation efficiency of Mix17 and normalized it to the efficiency of Tom40-3xHA immunoprecipitation, we found that virtually identical amounts of Mix17 were co-purified in presence and absence of the clogging construct (**Fig. 2 for the reviewers**).

We have removed a figure that was provided for the referees in confidence.

Fig. 2 for the reviewers. Arrested cytochrome b_2(167)ΔDHFR does not significantly influence the Mix17-Tom40 interaction.

© 2025. Published by The Company of Biologists under the terms of the Creative Commons Attribution License (https://creativecommons.org/licenses/by/4.0/).

Cytochrome b_2(167)ΔDHFR was treated with methotrexate to fold the DHFR domain and subsequently imported into mitochondria isolated from wild type yeast or a Tom40-3xHA expressing strain. Mitochondria were DSP treated, lysed in digitonin containing buffer and subjected to immunoprecipitation using anti HA-agarose. Omission of cytochrome b_2(167)ΔDHFR during the import reaction served as a control. Samples were analyzed by SDS-PAGE and immunoblotting. T: total lysate (4%); UB: unbound proteins (4%); B: bound proteins (100%). (Left) Immunoblotting of a representative experiment. (Right) Quantification of the experiment. Signal intensities were quantified using Image Studio software. The reduced immunoprecipitation efficiency of Tom40-3xHA after cytochrome b_2(167)ΔDHFR import was determined and the co-immunoprecipitation efficiencies of Mix17 and Tim23 were corrected. Values are shown as ratios of immunoprecipitation efficiencies with import of cytochrome b_2(167)ΔDHFR:without import of cytochrome b_2(167)ΔDHFR.

Although we think that this result is really interesting, we would prefer not to add it into the manuscript, as we probably did not occupy all TOM complexes with the clogging construct. Previous studies showed that only the import of TIM23 substrates is affected by cytochrome b_2(167)ΔDHFR clogging (Dekker et al., 1997). Thus, it might well be that Mix17 interacts with TOM complexes that do not interact with TIM23 complexes. We definitely need to analyze the effect of additional clogging constructs on the interaction of Mix17 and Tom40, before we are able to draw any final conclusion. Generation of these plasmids and the recombinant protein expression were unfortunately not possible during the time of this revision. We hope that reviewer 3 agrees that this is therefore beyond the scope of this study.

The authors state that the outer surface exposure of the Mix17 N-terminus is energy-dependent, however the experiment in Figure 1E shows quite a bit of cleaved Mix17 in intact mitochondria even after ATP depletion. Moreover, export of the N-terminus of Mix17 is not affected by the mitochondrial inner membrane potential, which is a prerequisite for ATP production. Can the authors clarify this apparent contradiction?
Finally, can they speculate on the putative molecular role of ATP in Mix17 export from the intermembrane space?
We thank the reviewer for the thoughtful comment. As we explained in the response to reviewer 2 point 1, we realized that the conditions determining the topology of Mix17 are far more complex than initially manifested. We feel that it is too early to attribute topology changes to ATP present in mitochondria and need more time to address this question. We plan to do this in an independent future study. Therefore, we removed the panel E from Fig. 1 (now Fig. 2) and made the respective changes in the text of the manuscript. We ask the reviewer for his/her understanding.

Minor point:

Figure 1C: Why is Tim50 fully degraded upon osmotic swelling, but only partially cleaved by trypsin in TX-100? Isn't this surprising?
We thank the reviewer for the important question and have now clarified the proteolytic accessibility assay in "Material and Methods". In the experiments shown in former Fig. 1 (new Fig. 2), mitochondrial membranes were lysed with TX-100 (lanes +TX), protease-treated and then directly TCA precipitated. Protease-resistant fragments of proteins are therefore TCA-precipitated and subsequently detected by SDS-PAGE and western-blotting. Protease K completely degrades Tim50 (Fig. 2B), whereas trypsin generates a specific Tim50 fragment (Fig. 2C). In contrast, mitochondria and mitoplasts are re-isolated by centrifugation after protease-treatment and then analyzed by SDS-PAGE and western-blotting. The protease-dependent soluble Tim50 fragment in the IMS is lost upon re-isolation of mitoplasts and, therefore, not detected (Fig. 1C; 3rd lane).

References

Burstein, S. R., F. Valsecchi, H. Kawamata, M. Bourens, R. Zeng, A. Zuberi, T. A. Milner, S. M. Cloonan, C. Lutz, A. Barrientos and G. Manfredi (2018). In vitro and in vivo studies of the ALS-FTLD protein CHCHD10 reveal novel mitochondrial topology and protein interactions. Hum Mol Genet *27*, 160-177. 10.1093/hmg/ddx397.

© 2025. Published by The Company of Biologists under the terms of the Creative Commons Attribution License (https://creativecommons.org/licenses/by/4.0/).

Chacinska, A., P. Rehling, B. Guiard, A. E. Frazier, A. Schulze-Specking, N. Pfanner, W. Voos and C. Meisinger (2003). Mitochondrial translocation contact sites: separation of dynamic and stabilizing elements in formation of a TOM-TIM-preprotein supercomplex. EMBO J *22*, 5370-5381.
Dekker, P. J., F. Martin, A. C. Maarse, U. Bomer, H. Muller, B. Guiard, M. Meijer, J. Rassow and N. Pfanner (1997). The Tim core complex defines the number of mitochondrial translocation contact sites and can hold arrested preproteins in the absence of matrix Hsp70-Tim44. EMBO J *16*, 5408-5419.
Kulak, N. A., G. Pichler, I. Paron, N. Nagaraj and M. Mann (2014). Minimal, encapsulated proteomic-sample processing applied to copy-number estimation in eukaryotic cells. Nat Methods *11*, 319-324. 10.1038/nmeth.2834.
Mokranjac, D., D. Popov-Celeketic, K. Hell and W. Neupert (2005). Role of Tim21 in Mitochondrial Translocation Contact Sites. J. Biol. Chem. *280*, 23437-23440.
Nagaraj, N., N. A. Kulak, J. Cox, N. Neuhauser, K. Mayr, O. Hoerning, O. Vorm and M. Mann (2012). System-wide perturbation analysis with nearly complete coverage of the yeast proteome by single-shot ultra HPLC runs on a bench top Orbitrap. Mol Cell Proteomics *11*, M111 013722. 10.1074/mcp.M111.013722.
Picotti, P., B. Bodenmiller, L. N. Mueller, B. Domon and R. Aebersold (2009). Full dynamic range proteome analysis of S. cerevisiae by targeted proteomics. Cell *138*, 795-806. 10.1016/j.cell.2009.05.051.
Shiota, T., K. Imai, J. Qiu, V. L. Hewitt, K. Tan, H. H. Shen, N. Sakiyama, Y. Fukasawa, S. Hayat, M. Kamiya, A. Elofsson, K. Tomii, P. Horton, N. Wiedemann, N. Pfanner, T. Lithgow and T. Endo (2015). Molecular architecture of the active mitochondrial protein gate. Science *349*, 1544-1548. 10.1126/science.aac6428.
Shiota, T., H. Mabuchi, S. Tanaka-Yamano, K. Yamano and T. Endo (2011). In vivo protein-interaction mapping of a mitochondrial translocator protein Tom22 at work. Proc Natl Acad Sci U S A *108*, 15179-15183. 10.1073/pnas.1105921108.
Waegemann, K., D. Popov-Celeketic, W. Neupert, A. Azem and D. Mokranjac (2015). Cooperation of TOM and TIM23 complexes during translocation of proteins into mitochondria. J Mol Biol *427*, 1075-1084. 10.1016/j.jmb.2014.07.015.

Second decision letter

MS ID#: jcs.263661R1

MS Title: The Mia40 substrate Mix17 exposes its N-terminus to the cytosolic side of the mitochondrial outer membrane

Authors: Moritz Resch; Johanna S Frickel; Korbinian Dischinger; Rachel Shen Wen Choo; Kai Hell; Max E Harner

Article Type: Research Article

Dear Dr Harner,

I am happy to tell you that your manuscript has been accepted for publication in Journal of Cell Science, pending standard publication integrity checks.

© 2025. Published by The Company of Biologists under the terms of the Creative Commons Attribution License (https://creativecommons.org/licenses/by/4.0/).
